# Supplementary material for: Magnetic and Magnetostrictive Behaviors of Laves-Phase Rare-Earth—Transition-Metal Compounds Tb1−xDyxCo1.95
Source: Materials (Basel). 2022 May 29;15(11):3884. doi: 10.3390/ma15113884 (PMC9182151; doi:10.3390/ma15113884)
Supplement: Supplementary file 1 [file materials-15-03884-s001.zip › materials-1733158-supplementary.pdf]

Figure S1

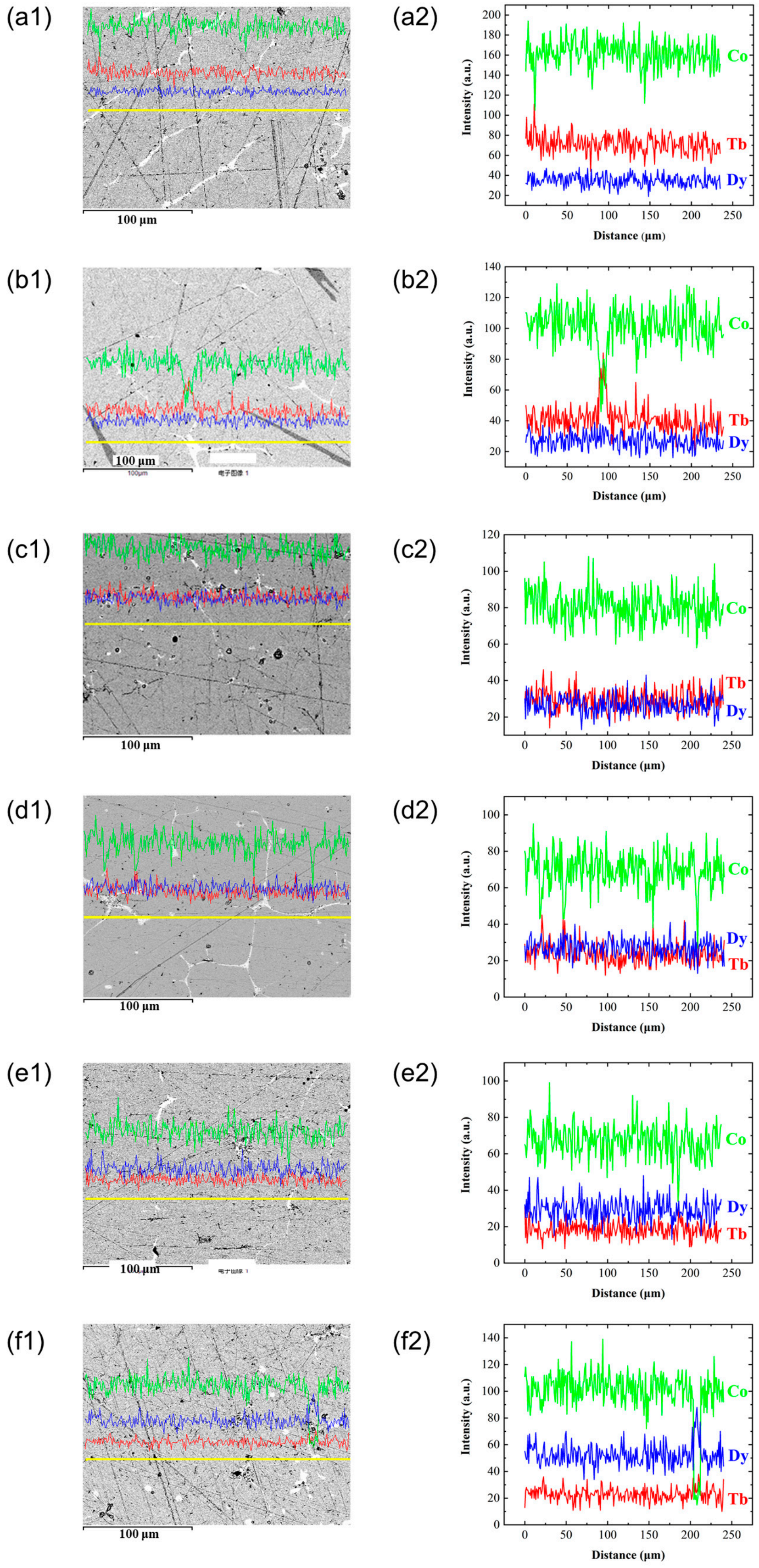

Figure S1. (**a1**–**f1**) The line scans of chemical elements for $Tb_{1-x}Dy_xCo_{1.95}$ alloys (x=0.3, 0.4, 0.5, 0.6, 0.7, 0.8), and (**a2**–**f2**) the corresponding element distribution (Tb, Dy, Co) for each composition.

Figure S2

(a)

$Tb_{0.7}Dy_{0.3}Co_{1.95}$

Elements	Relative mass percentage (exp.) [%]	Relative mass percentage (cal.) [%]
Tb	50.62	40.5
Dy	20.7	17.7
Co	28.71	41.8

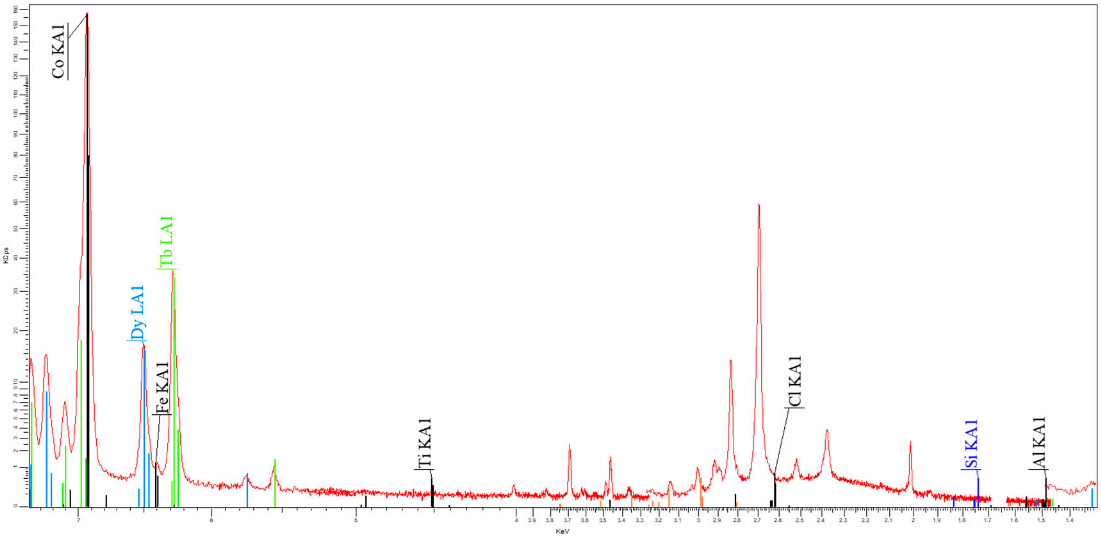

(b)

$Tb_{0.6}Dy_{0.4}Co_{1.95}$

Elements	Relative mass percentage (exp.) [%]	Relative mass percentage (cal.) [%]
Tb	40.52	34.64
Dy	26.8	23.61
Co	32.64	41.75

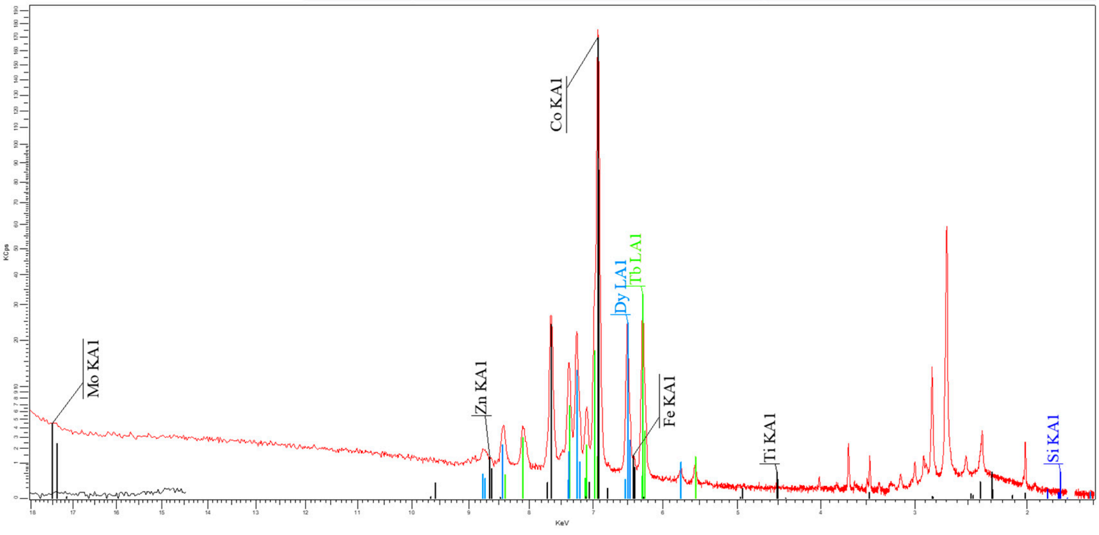

(c)

$Tb_{0.5}Dy_{0.5}Co_{1.95}$

Elements	Relative mass percentage (exp.) [%]	Relative mass percentage (cal.) [%]
Tb	35.6	28.83
Dy	34	29.48
Co	30.39	41.69

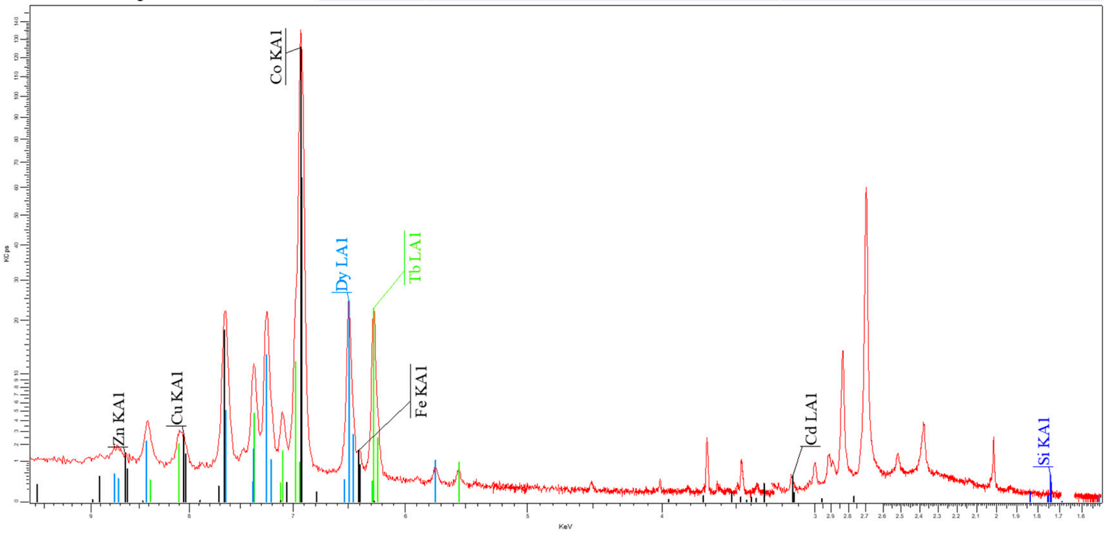

(d)

$Tb_{0.4}Dy_{0.6}Co_{1.95}$

Elements	Relative mass percentage (exp.) [%]	Relative mass percentage (cal.) [%]
Tb	25.38	23.03
Dy	36.96	35.33
Co	37.66	41.64

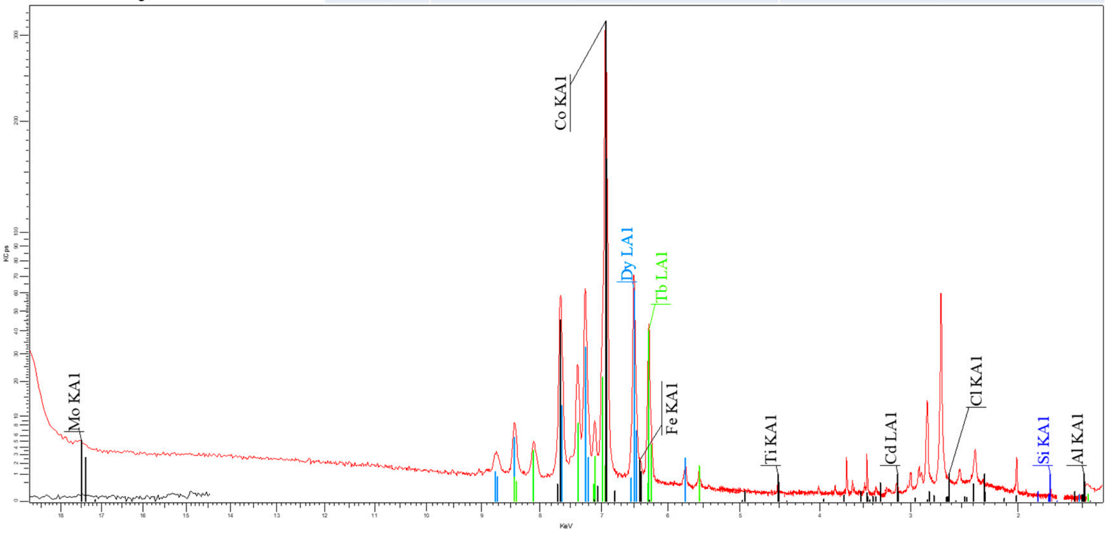

(e)

$Tb_{0.3}Dy_{0.7}Co_{1.95}$

Elements	Relative mass percentage (exp.) [%]	Relative mass percentage (cal.) [%]
Tb	19.2	17.24
Dy	43.27	41.16
Co	37.58	41.58

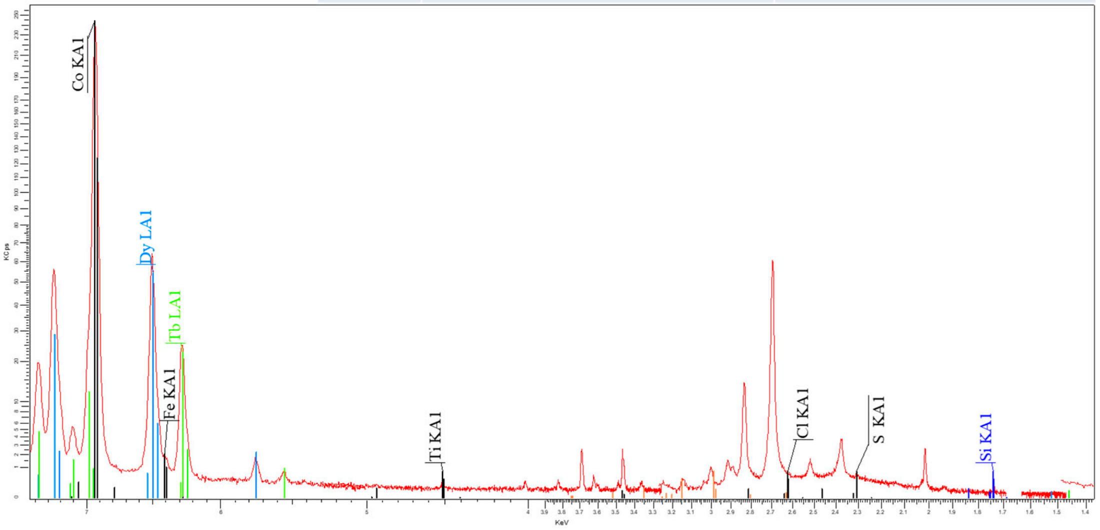

(f)

$Tb_{0.2}Dy_{0.8}Co_{1.95}$

Elements	Relative mass percentage (exp.) [%]	Relative mass percentage (cal.) [%]
Tb	14.3	11.49
Dy	54.5	46.98
Co	31.24	41.53

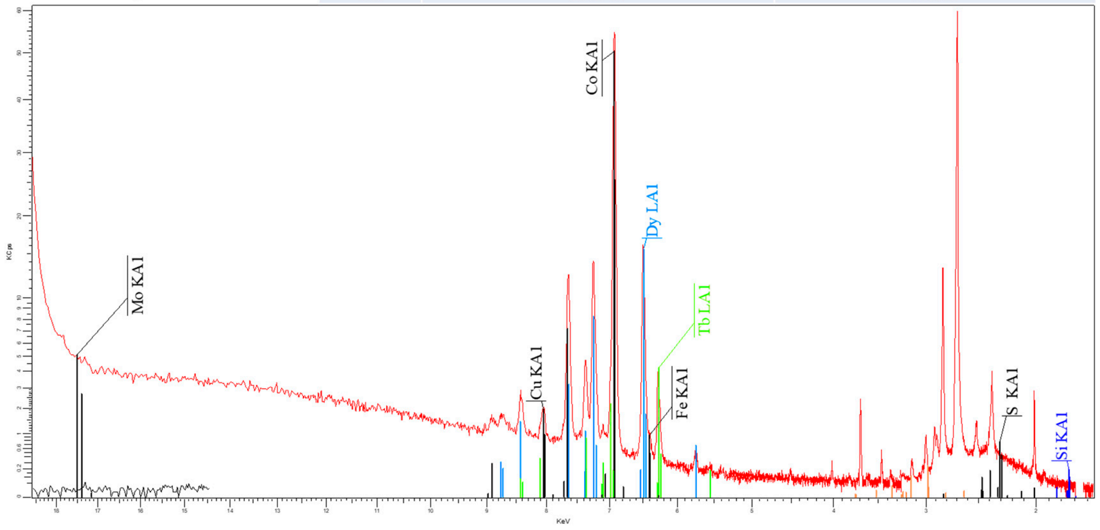

Figure S2. (**a**–**f**) The X-ray Fluorescence (XRF) results for $Tb_{1-x}Dy_xCo_{1.95}$ alloys (x=0.3, 0.4, 0.5, 0.6, 0.7, 0.8).

Figure S3

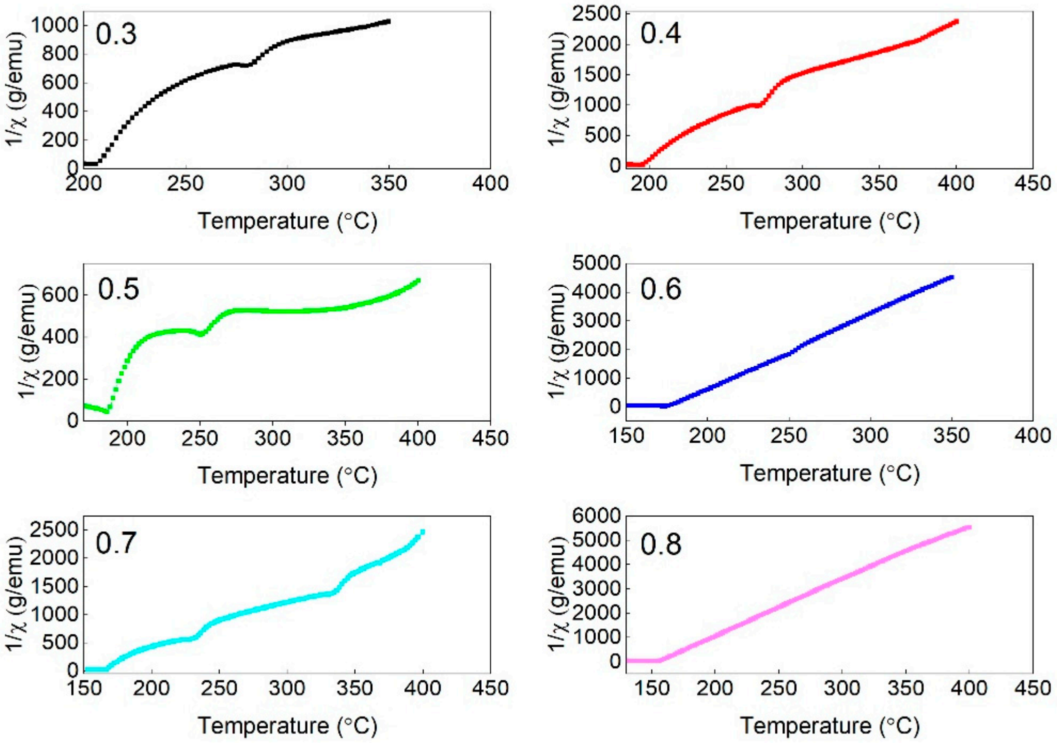

Figure S3. The inverse susceptibility 1/χ versus temperature curves above T_C for $Tb_{1-x}Dy_xCo_{1.95}$ alloys (x=0.3, 0.4, 0.5, 0.6, 0.7, 0.8).
